# Evaluation of neutrophil activation marker in patients with vitiligo

**DOI:** 10.1007/s11845-024-03777-4

**Published:** 2024-08-26

**Authors:** Hala M. El-Sadek, Asmaa A. Elmadbouly, Basma E. M. Risha

**Affiliations:** 1 Department of Dermatology, Faculty of Medicine for Girls, Al-Azhar University, Cairo, Egypt; 2 Department of Clinical Pathology, Faculty of Medicine for Girls, Al-Azhar University, Cairo, Egypt

**Keywords:** Calprotectin, Inflammation, Neutrophil activation marker, Vitiligo

## Abstract

**Background:**

Vitiligo is an inflammatory, autoimmune disorder. Its pathogenesis is unclear. A neutrophil activation marker (calprotectin) is a protein complex present in many different types of cells and may be used as an indicator of inflammation.

**Aims:**

This study is to assess calprotectin levels in non-segmental vitiligo patients and compare them to the severity of the illness to identify potential associations.

**Methods:**

The present inquiry was conducted on thirty non-segmental vitiligo patients and thirty healthy volunteers matched in terms of age and gender. The Vitiligo Area Scoring Index was used to assess the vitiligo severity. Calprotectin levels were measured in serum samples obtained from all participants by Enzyme Linked Immunosorbent Assay.

**Results:**

Compared to controls, non-segmental vitiligo patients had considerably elevated serum calprotectin levels. Additionally, calprotectin levels were shown to have a significant positive association with disease severity (*r* = 0.833, *P* = 0.000).

**Conclusions:**

Elevated levels of serum calprotectin in non-segmental vitiligo patients relative to healthy individuals with high sensitivity indicated that it may have a role in the vitiligo pathophysiology and can act as a marker for disease monitoring.

## Introduction

Vitiligo is a pigmentary disorder with an unknown cause that is identified by the targeted destruction of pigment producing cells, which diminishes the pigment in the skin's affected regions. The typical lesion is a completely amelanotic, non-scaly, milky-white macule with clearly defined borders [1]. It is a rare skin condition, affecting 0.4% to 2.0% of the global population, with females being slightly more likely to develop it, with half of cases starting in childhood [2]. Chronic vitiligo affects social and occupational functioning, causing distress, shame, and embarrassment, leading to depression and anxiety in sufferers [3].

The pathophysiology of vitiligo is intricate, involving genetic predisposition, oxidative stress, melanocyte auto-aggressiveness, and immunological tolerance mechanisms, with the exact causes still not fully understood. Vitiligo is a condition influenced by a combination of innate and adaptive immune system elements, metabolic factors, melanocyte adhesion to epithelium, and genetic factors [4].

Genetic factors contribute to the development of vitiligo, including changes in genes such as FOXP3, TSLP, NALP1, and HLA alleles. Additionally, oxidative stress increased reactive oxygen species release and deficiencies of antioxidant in vitiligo patients skin leads to melanocyte undergo mitochondria-dependent apoptosis. On another hand melanocyte destruction or suppression of melanin synthesis may also be caused by neural processes involving neurochemical mediators [5].

The existence of antibody-mediated auto reactive CD8 + T cells, which cause melanocytes to be destroyed, supports the autoimmune origin of vitiligo. Likewise, it is believed that memory T cells stay and have a role in the lesions' recurrence. Based on the latest findings, regulatory T cells may aid in vitiligo by boosting the immune response [6].

The inflammatory chemokines’ including CXCL9, CXCL10, and CXCL12 are Th1-related chemokines play a crucial role in the recruitment and activation of cytotoxic CD8 + T cells in inflammatory tissues. The optimal synthesis of IFNγ and TNFα by lymphocytes triggers the release of CXCL10. When this chemokine attaches to the cell's CXCR3 receptor, it activates and increases the recruitment of T lymphocytes, starting an inflammatory cascade that ultimately leads to the demise of melanocytes [7].

In response to external stimulation, keratinocytes release the S100A protein, which functions as a modulator of the transfer of epidermal responses and is known to cause activated CD8 + T lymphocytes to exert their cytotoxic effect [8]. Moreover, it increases the synthesis of inflammatory cytokines like TNF-α, IL-6, and IL-1β as well as stimulation of the Nucleotide-binding Oligomerization Domain-Leucine Rich Repeat (NOD-LRR), Pyrin domain-containing Protein (NLRP3), and NFăB. Additionally, under stressful situations, S100A regulates the chemokine CXCL16, which draws CD8 + T lymphocytes and enhances the keratinocyte and melanocyte counts [9].

After identifying antigen (Ag)-MHC, CTLs contribute to keratinocyte, basal epithelial cell, and melanocyte apoptosis induction through three primary pathways: granzyme, FAS–FAS ligand (FASL), and TNF-TNFR [10]. The apoptotic cascade is initiated when FASL (CD178) binds to FAS (CD95) on basement membrane, melanocyte, and keratinocyte cells. The Fas-associated protein with death domain (FADD) cytosolic adapter, which is attached to caspase-8 and initiates a death-inducing signaling complex (DISC), is recruited via the FAS-FASL interaction. Caspase-3 is activated by the DISC complex, leading to the induction of caspase-activated DNase120 that breaks down DNA when it reaches the cell nucleus [11]. Calprotectin (CLP) is a pro-inflammatory heterodimeric compound consisting of two proteins that bind to calcium named S100A8 and S100A9, members of the S-100 protein family [12].

CLP is found in a wide range of human organs, body fluids, and cells, and it is particularly prominent in regeneration cells like macrophages, neutrophils, monocytes, endothelium, and epithelial cells [13].

Calprotectin is a key marker for neutrophil activation and inflammation, since the correlation between its release from neutrophils and inflammatory reactions; it can serve as a useful biomarker for a number of illnesses, such as autoimmune disorders and infections. In response to inflammatory conditions, neutrophil granulocytes produce serum calprotectin. This substance’s production has been linked to anti-inflammatory and anti-infective properties such scavenging of reactive oxygen species, chemotaxis, chelation of divalent cations, regulation of myelopoiesis, and direct antimicrobial activity [14].

The S100A8/S100A9 complex facilitates the coordination of an inflammatory response by regulating the intracellular pathways of innate immune cells. CLP promotes leucocyte recruitment by modifying cytoskeletal rearrangements and makes it easier for arachidonic acid to reach inflammatory areas [15]. It has cytotoxic and apoptotic properties, impacting cell proliferation by triggering caspase-3-dependent apoptosis and blocking different cell types’ growth, such as lymphocytes, fibroblasts, bone marrow cells, and macrophages, at high concentrations [16]. Additionally, CLP stimulates the production of pro- and anti-inflammatory cytokines; following treatment with S100A9, human monocytes produced IL-6, IL-1β, and TNF-α in response to oxidative stress [17].

Given all we know, CLP has been researched in a number of autoimmune illnesses; nevertheless, serum CLP has not been studied in vitiligo; therefore, the goal of this research was to determine the association among serum CLP levels and vitiligo, and investigate potential correlations with disease severity.

## Subjects and methods

In this study, thirty patients with non-segmental vitiligo (NSV) who were identified based on clinical and dermoscopic characteristics were compared to thirty age-and sex-matched healthy participants as controls. All subjects were chosen from Dermatology Outpatient Clinic of Al-Zahraa University hospital. The Research Ethics Committee of Al-Azhar University in Cairo, Egypt, provided its approval to the study, approval number (2023082067). Written informed permission was provided by each participant prior to participating in the research. Individuals younger than 18 years, those suffering from autoimmune or inflammatory disorders systemic lupus erythematous, microbiological infections, neoplastic conditions, Crohn’s disease, ulcerative colitis, rheumatoid arthritis, pregnancy, and cystic fibrosis that may affect calprotectin level all were excluded from the study.

### Demographic and clinical evaluation

All subjects underwent a thorough history and dermatological clinical evaluation, including checks of skin, scalp, mucous membranes, and nail apparatus. Patients with vitiligo were assessed using the Vitiligo Area Scoring Index (VASI). The VASI for each body region is calculated by combining vitiligo area and depigmentation extent within each hand unit, which represents 1% of the body’s surface area, full depigmentation 100% with no pigment present; 90% of pigment flecks are visible; 75% more area is depigmented than pigmented; 50% of locations with pigmentation and those without; 25% of the area is pigmented; and 10% depigmented with only a few spots [18].

### Laboratory investigations

For each participant, three milliliters of venous blood were taken and centrifuged for serum separation; sera were kept at − 80 °C until the time of assay.

### Measurement of CLP serum levels

Serum calprotectin levels were measured using the commercially available human ELISA kit, supplied by Sun Red, Shanghai, China, Lot number (202,401), Catalog number (201–12-5461B). Using an automated ELISA system, according to the manufacturer’s instructions, the assays were done from both control and patients.

### Statistical analysis

Following collection, revision, coding, and entry, the data were loaded into a statistical package for social science (IBM SPSS) version 26. When the data were determined to be non-parametric, the quantitative data were reported as the median, inter-quartile range (IQR), and mean, standard deviations, and ranges. Quantitative variables were also shown as percentages and numbers. The comparison between groups with qualitative data was done by using Chi-square test. Two independent groups with parametric distribution and quantitative data were compared using the independent t-test; the Mann–Whitney test was used for non-parametric distributions. Spearman correlation coefficients were utilized to assess the correlation between two quantitative factors within the same group. Using the receiver operating characteristic curve (ROC) and its sensitivity, specificity, positive predictive value (PPV), negative predictive value (NPV), and area under the curve (AUC), to differentiate between the patient and control groups, the ideal CLP level cutoff value was established. We utilized a 95% confidence interval. As a result, the significance threshold of the *p* value was set at < 0.05.

## Results

Thirty non-segmental vitiligo patients and thirty healthy subjects were involved in this case–control study. Of the thirty vitiligo patients, fourteen (46.7%) were males and ranged in age from 18 to 52 years, with a mean age of 37.43 ± 10.01 years. The vitiligo median duration was 6.5 years, with a range of 1 to 12 years (3–10). Severity of disease ranged from 20 to 85 with a mean of 48.2 ± 18.2 (Table 1).

**Table 1:** Demographic data characteristics of the studied patients

		Patients group (no. = 30)
Age		Mean ± SD
		Range
Sex		Male
		Female
Duration (year)		Median (IQR)
		Range
Family History	No Yes	28 (93.3%) 2 (6.7%)
VASI score	Mean ± SD Range	48.2 ± 18.2 20–85
Calprotectin (ug /ml)	Median (IQR) Range	246.1 (214.4–308.2) 165.4–2400

Regarding to the CLP serum levels, a highly statistically significantly elevation in serum CLP levels were found in vitiligo patients’ in contrast to healthy individuals (*p* < 0.001). Nonetheless, no statistically significant differences between age and sex in both groups were reported (*p* > 0.05) (Table 2).

**Table 2:** Comparison between cases and control groups regarding demographic data and calprotectin level

		Control group	Patients group	Test value	*P*value	Sig
		No. = 30	No. = 30			
Age	Mean ± SD	35.77 ± 10.45	37.43 ± 10.01	− 0.631•	0.531	NS
	Range	18–50	18–52			
Sex	Male Female	10 (33.3%) 20 (66.7%)	14 (46.7%) 16 (53.3%)	111.1*	0.292	NS
Calprotectin (ug/ml)	Median (IQR) Range	195.55 (162.2–264.1) 40.1–384.5	246.1 (214.4–308.2) 165.4–2400	− 2.758≠	0.006	HS

*P *value > 0.05, non-significant; *P* value < 0.05, significant; *P* value < 0.01, highly significant. *Chi-square test;  •Independent t-test; ≠ Mann-Whitney test

**Fig. 1:**
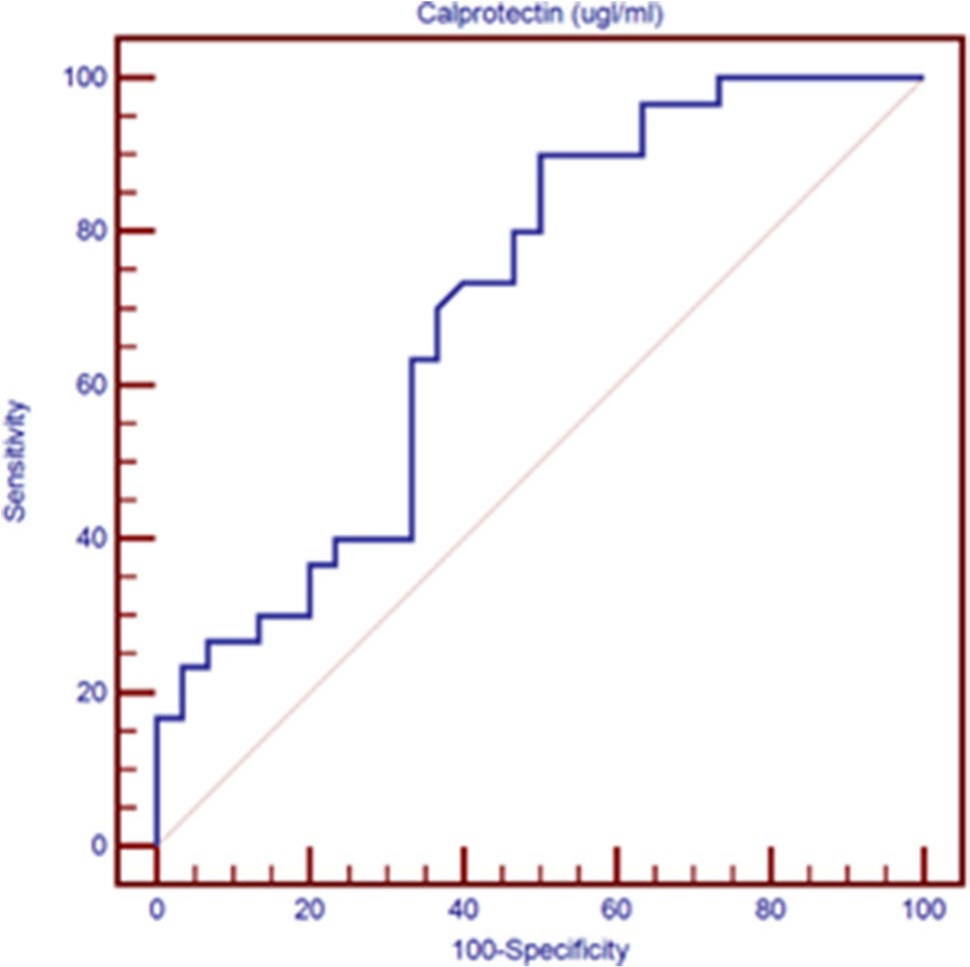
Receiver operating characteristic curve (ROC) for calprotectin level to differentiate between patients group and control group

The Roc curve showed that serum CLP levels had a sensitivity of 90% and specificity of 50.00%, at cut-off level of > 184.5 ug /ml **(**Fig. 1).

In relation to CLP levels and disease severity, a highly significant positive correlation was found among CLP levels and VASI score (*r* = 0.833, *P* = 0.000) **(**Table 3).

**Table 3:** Correlation of CLP levels with age, duration, VASI score, sex, and family history among patients group

Patients group	Calprotectin (ugl/ml)	
	*R*	*P*value
Age	− 0.230	0.222
Duration (year)	− 0.222	0.239
VASI-score	0.833**	0.000

*P* value > 0.05, non-significant;* P* value < 0.05, significant; *P* value < 0.01, highly significant, Spearman correlation coefficient •Mann-Whitney test; ≠ Kruskal-Wallis test

## Discussion

Vitiligo is a long-term, acquired illness that causes immunological aggression in melanocytes, leading to skin and mucosal membranes develop hypo- or a chromic macules and patches, and the condition may coexist with systemic conditions like sensorineural deafness, thyroiditis, and uveitis [4].

Numerous researches have looked at the connection among serum CLP levels and particular inflammatory conditions, like psoriasis, acne vulgaris, and atopic dermatitis, where elevated CLP levels have been observed [19–21]. However, no reports exist about the association between vitiligo and serum CLP levels. Psoriasis, atopic, and acne are autoimmune conditions; autoimmune diseases like vitiligo may share similar pathogenesis in epidemiological and genetic studies.

Certain S100 proteins have a significant part in the pathogenesis of autoimmune and inflammatory illnesses via stimulating antigen-presenting cells, innate immunity, and adaptive immunological responses [22].

Crucially, our research revealed for the initial time that NSV patients had noticeably higher levels of the serum CLP compared to healthy controls. Furthermore, the ROC curve demonstrated that the CLP levels have a high sensitivity for differentiating between patients and healthy controls indicated that CLP might play a part in the pathophysiology of vitiligo; this may be due to its possible engagement in the inflammatory and autoimmune process related with vitiligo. In agreement with Kaiqiao He et al. [23] who revealed that NSV patients had significantly higher circulating levels of S100A9, he showed excellent accuracy in assessing disease activity. Additionally, a research by Elwan et al. [20] on psoriasis found that a ROC analysis that CLP serum levels have 75% sensitivity and 69% specificity.

S100A9 interacts with TLR-4-expressing cells, promoting pro-inflammatory signaling cascade. It creates heterodimeric compounds with S100A8, forming heterotetramers (CLP) in calcium. Restricting CLP activity by inhibiting a particular TLR4-binding region can reduce systemic side effects and provide specific anti-inflammatory therapy [24]. Investigating CLP role and intervention method in vitiligo occurrence is crucial.

It has been reported that S100 proteins are linked to autoimmune illnesses due to their role in inflammation, triggering the release of adhesion molecules, chemokines, and cytokines after binding to their receptors on target cells leads to increased leukocyte recruitment, infiltration, and tissue damage [25].

Stascheit et al. [26] observed that CLP is a marker of the severity of a variety of disorders, such as inflammatory bowel diseases, acne, atopic dermatitis, psoriasis, myasthenia gravis, and rheumatoid arthritis.

Consistent with the results of Fouda et al. [21] who demonstrated a substantial positive correlation between calprotectin levels and the severity of acne, our research results also revealed a noteworthy positive link among vitiligo severity and CLP serum levels. Furthermore, in psoriatic patients, there was a strong association found by previous studies [20, 27] between the severity of psoriasis and serum levels of CLP. Additionally, Ali et al. [19] discovered a significant correlation between atopic patients’ blood CLP level and severity of disease. This was in contrast to the findings of Korkmaz and Fıçıcıoğlu [28], who showed no significant connection between the severity of acne and serum CLP level.

The current study found that serum CLP levels showed no significant correlation with patient age and sex, family history, or duration of disease (*p* > 0.05 for each). Our findings are in consistent with Elwan et al. [20] who found no significant relationship between sex and psoriasis duration. Furthermore, Ali et al. [19] reported a non-significant association was shown between the serum CLP level of atopic patients and the patients’ age and disease duration.

In contrast to Fouda et al. [21], they indicated a strong positive relationship between the CLP serum levels and the period of acne.

In light of the findings of our inquiry, we suggest that calprotectin could be a useful indicator of vitiligo severity and a useful tool for assessing how well a treatment is working. Calprotectin, potentially involved in inflammatory processes, may be a potential target for vitiligo treatment, though definitive conclusions are yet to be drawn.

The small sample size, lack of further fecal specimens for CLP examination, and lack of follow-up of CLP levels in vitiligo patients who received therapy thereafter are among the study’s limitations and should be highlighted in future research.

## Conclusions

This is the initial study to evaluate the CLP serum levels in patients with vitiligo. Given that the concentrations of CLP in the vitiligo patients were considerably greater in comparison to the healthy subjects. The study found a strong positive correlation between disease severity and CLP levels, suggesting that CLP can serve as a useful indicator of vitiligo severity and course. To clarify the relationship between CLP and the pathophysiology of vitiligo, more research is needed.

## Data Availability

The data that support the findings of this study are available upon reasonable request.
